# Recognition and Counting of Apples in a Dynamic State Using a 3D Camera and Deep Learning Algorithms for Robotic Harvesting Systems

**DOI:** 10.3390/s23083810

**Published:** 2023-04-07

**Authors:** R. M. Rasika D. Abeyrathna, Victor Massaki Nakaguchi, Arkar Minn, Tofael Ahamed

**Affiliations:** 1 Graduate School of Science and Technology, University of Tsukuba, Tennodai 1-1-1, Tsukuba 305-8577, Japan; 2 Department of Agricultural Engineering, University of Peradeniya, Kandy 20400, Sri Lanka; 3 Department of Agricultural Engineering, Yezin Agricultural University, Nay Phi Taw 150501, Myanmar; 4 Faculty of Life and Environmental Sciences, University of Tsukuba, Tennodai 1-1-1, Tsukuba 305-8577, Japan

**Keywords:** dynamic accuracy, localization, YOLO, Deep SORT, fruit detection

## Abstract

Recognition and 3D positional estimation of apples during harvesting from a robotic platform in a moving vehicle are still challenging. Fruit clusters, branches, foliage, low resolution, and different illuminations are unavoidable and cause errors in different environmental conditions. Therefore, this research aimed to develop a recognition system based on training datasets from an augmented, complex apple orchard. The recognition system was evaluated using deep learning algorithms established from a convolutional neural network (CNN). The dynamic accuracy of the modern artificial neural networks involving 3D coordinates for deploying robotic arms at different forward-moving speeds from an experimental vehicle was investigated to compare the recognition and tracking localization accuracy. In this study, a Realsense D455 RGB-D camera was selected to acquire 3D coordinates of each detected and counted apple attached to artificial trees placed in the field to propose a specially designed structure for ease of robotic harvesting. A 3D camera, YOLO (You Only Look Once), YOLOv4, YOLOv5, YOLOv7, and EfficienDet state-of-the-art models were utilized for object detection. The Deep SORT algorithm was employed for tracking and counting detected apples using perpendicular, 15°, and 30° orientations. The 3D coordinates were obtained for each tracked apple when the on-board camera in the vehicle passed the reference line and was set in the middle of the image frame. To optimize harvesting at three different speeds (0.052 ms^−1^, 0.069 ms^−1^, and 0.098 ms^−1^), the accuracy of 3D coordinates was compared for three forward-moving speeds and three camera angles (15°, 30°, and 90°). The mean average precision (mAP@0.5) values of YOLOv4, YOLOv5, YOLOv7, and EfficientDet were 0.84, 0.86, 0.905, and 0.775, respectively. The lowest root mean square error (RMSE) was 1.54 cm for the apples detected by EfficientDet at a 15° orientation and a speed of 0.098 ms^−1^. In terms of counting apples, YOLOv5 and YOLOv7 showed a higher number of detections in outdoor dynamic conditions, achieving a counting accuracy of 86.6%. We concluded that the EfficientDet deep learning algorithm at a 15° orientation in 3D coordinates can be employed for further robotic arm development while harvesting apples in a specially designed orchard.

## 1. Introduction

**Figure 1:**
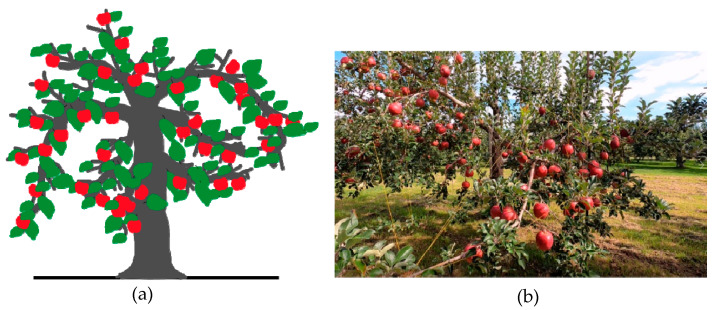
(**a**) Conventional tree structure with complex distribution of branches and (**b**) real conventional apple orchard conditions (Aomori Prefectural Industrial Research Center, Research institute in Kuroishi, Aomori Prefecture, Japan).

Although apples are the most widely grown fruits, apple growers frequently encounter difficulties when increasing production due to the scarcity of skilled and seasonal labor and quality defects during postharvest operations in conventional apple orchards. The conventional apple-harvesting system is a seasonal, labor-intensive, dependent system that increases farming costs. Robotic platforms are considered a promising solution to overcome these difficulties. Robotic platforms can replace manual labor, thus avoiding problems such as labor shortages during the picking season when fruits are handpicked. However, robotic platform implementation is also very challenging in conventional orchards because of their irregular shapes, sizes of plants, and distributions of apples in trees (**Figure 1**). The recognition of apples and harvesting in irregularly shaped apple orchards at dynamic speeds are additional challenges.

**Figure 2:**
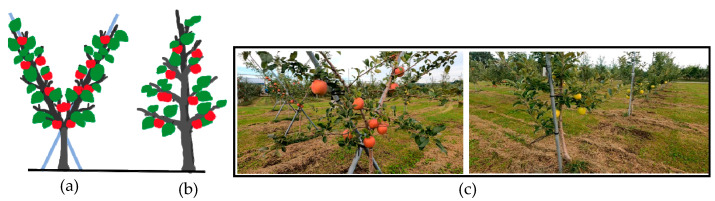
(**a**) V-shaped tree architecture, (**b**) tall spindle tree architecture, and (**c**) V-shape and spindle architecture in recent orchard practices considering automated system.

Because of the irregular shape of orchards, researchers have made significant efforts to achieve successful robotic harvesting with proper positional information of apples before harvesting. Estimation of the pose for each localized apple is the most important task of the vision system [1]. In the apple-picking cycle, localization, the manipulator reaching the target location, end effector detachment, and final collection of harvested fruit are the main components [2]. Inaccurate localization can damage the apples as well as the manipulator since the algorithms determine the manipulator collision avoidance path to the fruit and grasping orientation after localization [3]. Therefore, a difficult task in orchard automation is the provision of correct information about apple location to a robot harvester or an end effector. In providing location information, dense canopies, branches, and apple clusters hinder the estimation of positional accuracy in vision systems that use stereo cameras. Moreover, once accurate coordinates for apple grasping are identified, it is possible to avoid damages to leaves and branches by using an end effector along with tactile or ultrasonic sensors. Furthermore, environmental factors such as sunlight highly affect the stereo-perception-based vision system, creating problems in estimating the target movement of manipulators for grasping apples from complex orchard environments. Therefore, accurately locating apples is even more challenging considering the recent developments in apple harvesters [4]. Specially developed orchards and environmental infrastructure have the potential to aid the deployment of robotic harvesting systems. Specially designed apple orchards can contribute to the success of end effectors or manipulators with smaller degrees of freedom at higher operational speeds using machine vision systems (**Figure 2**).

The development of computer vision and the advancement of electronics provide immense opportunities for agriculture farmers in yield mapping, yield prediction, and harvesting, allowing a smooth flow of agriculture products from farms to consumers [5,6]. In addition to the architecture of the orchard environment and dataset training, efficient algorithms for counting are also very important in precision orchard management [7,8]. Training datasets from those augmented orchard architecture systems are critical for apple recognition and counting by robotic systems.

Occlusion by leaves and branches is the main problem hindering the performance of the vision systems of robotic platforms. Furthermore, the yield estimation of apples is also affected due to miscounting of apples. Different studies have been carried out on the recognition of apples and distinguishing of leaves using multispectral dynamic images by extracting texture features [9]. The random sample consensus (RANSAC) algorithm was applied to localize apples and measure the diameters of each detected apple, which aids end effector operations [10] by helping the manipulator to avoid occlusions. Changes in the illumination level is another factor that affects the accurate localization of apples. Previous studies have been carried out on methods for avoiding illumination conditions, and a color-based detection algorithm was developed with the Flash-No-Flash illumination protocol for sweet pepper harvesting [11,12]. Recent studies show that CNN-based deep learning combined with advanced semantic expression can be used to autonomously learn depth features [13]. A mask-region-based convolutional neural network (Mask R-CNN) for strawberries has been proposed for detecting target fruits, followed by the Mask R-CNN for the identification of picking points [14]. Moreover, a guava fruit detection and 3D pose estimation algorithm was developed based on Euclidean clustering using an RGB-D camera [15]. The above studies have made significant contributions in this area; however, conventional orchard systems complicate apple recognition by robotic platforms. In a conventional orchard system, occlusion of leaves and fruit positional information also presents a challenge due to sunlight obstruction in dense canopies. Specially designed infrastructure of apple orchards incorporating concepts such as tall spindle, V-shape, Espalier, and step-over espalier fruit training systems has the potential to enhance accurate recognition and counting of apples to achieve successful robotic operational platforms. An automated video-processing-based fruit-counting method has been developed for a vertical fruiting wall apple orchard based on tracking tree trunks. YOLOv4-tiny was combined with a channel spatial reliability–discriminative correlation filter (CSR-DCF). The mAP@0.5 was reported to be 99.35% for detecting trunks, and the counting accuracy was 91.49% [7].

Furthermore, deep learning algorithms and their recent backbone developments in CNN systems with large datasets can be trained to attain higher recognition accuracy. An RGB-D camera with trained (RGB) datasets can be used to develop vision systems for end effectors as an approach to targeting apples for harvesting in different orientations.

Therefore, the objective of this research was to develop a recognition and apple-counting system using deep learning algorithms based on an RGB-D D455 camera from specially designed orchard datasets for robotic harvesting systems.

## 2. Related Work

### 2.1. Visual Perception of Apples

The development of fast electronics has led to more opportunities to develop different neural networks and sensors that can be utilized for different applications. The same technology is employed for fruit detection and localization [2]. Almost all robotic manipulation in real time starts with the detection and localization of the target object. In orchard robotics for fruit, branch, and leaf detection, many studies have been carried out using RGB cameras to detect the target locations [16,17].

Hyperspectral, multispectral, RGB, and thermal cameras have been used for fruit detection; in the case of thermal cameras, they use different thermal inertia to differentiate fruits from the background with ambient temperature differences [18]. LiDAR uses the time-of-flight (ToF) principle that is applied for 3D crop models [19]. Additionally, LiDAR can measure reflectance differences; for example, Fuji apples can be distinguished from the tree trunk and leaves with 85% success with this method [20]. A study was performed with a Kinect sensor for the localization of bicolored and red apples to obtain color and depth information, and the processing time was less than 1 s for 20 apples [10]. Accurate fruit localization is a must during robotic fruit harvesting in orchards. Another study was carried out using a forward-looking infrared (FLIR) camera integrated with dynamic imaging (MSX) technology, resulting in a recognition precision greater than 92% for complete fruits and a recognition precision greater than 72% for fruits with incomplete regions and a processing time of less than 1 s [9].

RGB-D cameras follow three basic principles: the first principle is the time of flight (ToF), the second principle is structured light (SL), and the third principle is active infrared stereo (AIRS). ToF-based cameras use an infrared-emitting mechanism, sense returning reflectance signals via a detector, and calculate the distance to target objects based on the travel time and the speed of light. The most important aspect of the ToF method is that the depth information does not change by the gray level and does not change based on the surface characteristics of the target object, resulting in accurate 3D images. Hence, variation in the distance of the target object does not affect the depth accuracy [21]. Realsense^©^ (Intel Corporation, Santa Clara, California, USA) D400 family cameras use active infrared stereo technology. Red, Green, and Blue Depth (RGBD) cameras provide accurate details for fruit detection and localization, which is the working principle of stereo triangulation [22]. RGBD cameras can be used to distinguish target fruits based on color, texture, and geometric shape with the help of deep neural networks [23,24].

Different techniques are applied for fruit detection and localization, and RGB images are essential because they can be combined with depth images, point cloud images, or infrared images [21]. For localization, depth or point cloud information is essential [12].

### 2.2. Apple Detection with RGB and Depth Images

RGB images provide information about fruit color, texture, and geometric shape. RGB images integrated with machine vision can be used to distinguish target fruits from branches and leaves. Previous studies have shown that using RGB images can lead to a high detection rate of greater than 80% [21]. Apple detection with an 80.8% success rate was achieved by Faster R-CNN object detection algorithm with VGG-16 convolutional neural network (CNN) on the backbone using RGB images taken by Kinect v2 [25]. RGB-D images have an extra feature, depth, which is incorporated into each pixel and forms a depth map [26]. These depth images can be easily used for detecting fruits. A study on tomato fruit detection and counting based on the Mask R-CNN algorithm (instance segmentation based) reported using a Realsense D435 camera in laboratory conditions. The results show that the Mask R-CNN has the potential to be used for learning objects according to their depth values [7]. Orchard images provide different details, such as fruit quality, diseases, crop load, fruit nutrition states, and the maturity levels of fruits, and these data can be employed for the automation of different orchard operations [27]. These images have been combined with deep learning methods for classification detection and segmentation. Deep neural networks (DNNs) need to be trained with a dataset that has to be manually labeled, while DNSs can detect orchard fruits with high accuracy [5]. For inferencing, real-time DNNs need high-performing graphic processing computations when utilized in visually guided agricultural applications to achieve good results.

Thus, different classifiers have been integrated with CNNs and observed for their ability to improve the performance of detection models. A study was carried out with a Kinect V2 camera sensor for citrus fruit detection and localization for coordinate transformation, and image classification was performed based on the Bayes classifier with depth filtering. Then, density clustering was performed, followed by the classifier, which is a geometry-based feature support vector machine, to eliminate false positives. The results illustrate an F1 score of 0.9197 and localization errors x, y, and z of 7.0 ± 2.5 mm and 4.0 ± 3.0 mm [28]. 

Moreover, fusion of different sensors can increase accuracy while reducing the noise from environmental parameters. Thus, fusion of LiDAR and a 3D camera was used for the detection of apples to achieve a high accuracy of localization, as a way of overcoming the variation in light conditions. This study followed target and no-target approaches based on two SOTA (state-of-the-art) extrinsic calibration methods to identify the extrinsic matrix between a 3D camera and LIDAR. The localization results show standard deviations of 0.245 cm, 0.227 cm, and 0.275 cm from distances of 0.5 m, 1.2 m, and 1.8 m using LiDAR and the 3D camera, respectively. Semantic segmentation was integrated with LiDAR data to examine the noisy point cloud, which was highly unstructured. The results show a higher accuracy in mIoU (86.2%) analyzing the high-resolution point clouds [13]. 

While considering all the limitations and challenges, this study was carried out using a D455 Realsense camera, with the following contributions:

- The use of a state-of-the-art algorithm combined with Deep SORT to detect, count, and track apples at three forward dynamic speeds and three different camera orientations to propose an optimal orientation for grasping apples while harvesting using an end effector or robotic arm.
- The use of a depth stream overlapped with an RGB stream to obtain depth values of detected apples and compare the accuracy of depth values in dynamic stages to identify the most suitable CNN model for deploying an end effector or robotic arm while harvesting apples at the recommended orientation.

## 3. Materials and Methods

### 3.1. Field Data Collection from a Specially Designed Orchard

GoPro Hero 10 (GoPro, Inc. Woodman Labs, Inc. U. San Mateo, CA, USA) was selected to collect data at the Apple Research Institute (Aomori Prefectural Industrial Research Center, Research Institute in Kuroishi, Aomori Prefecture, Japan). The videos were recorded at a resolution of 3840 × 2160 pixels on the 27th and 28th of September 2022, and videos were taken in the morning, noon, and evening to cover different daylight conditions while recording videos from different angles. The recording was performed from a height of 1 m to a height of 1.5 m from the ground, and the camera was placed 1 to 2 m from the trees.

### 3.2. Image Data Preparation for Data Training

**Figure 3:**
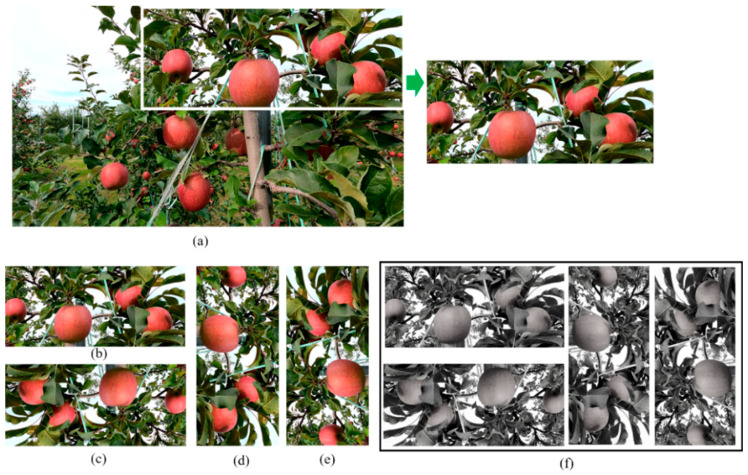
Dataset preparation and augmentation: (**a**) original RGB image, (**b**) cropped image from original image, (**c**) image rotated 180°, (**d**) image rotated 90° clockwise, (**e**) image rotated 90° counterclockwise, and (**f**) all images when converted to grayscale.

The recorded videos (dataset) were transformed into a set of images that were cropped, and image augmentation was performed to increase the accuracy of the dataset by applying different conditions. The cropped images were rotated 180° so that they were upside down, and then were rotated 90° clockwise and 90° counterclockwise, after which all the images were converted to grayscale (**Figure 3**). The original number of images was 800; however, after performing the augmentation, the total number of images increased to 6511. After augmentation of the images, annotation was performed using a self-coding Python program based on a YOLO format.

**Figure 4:**
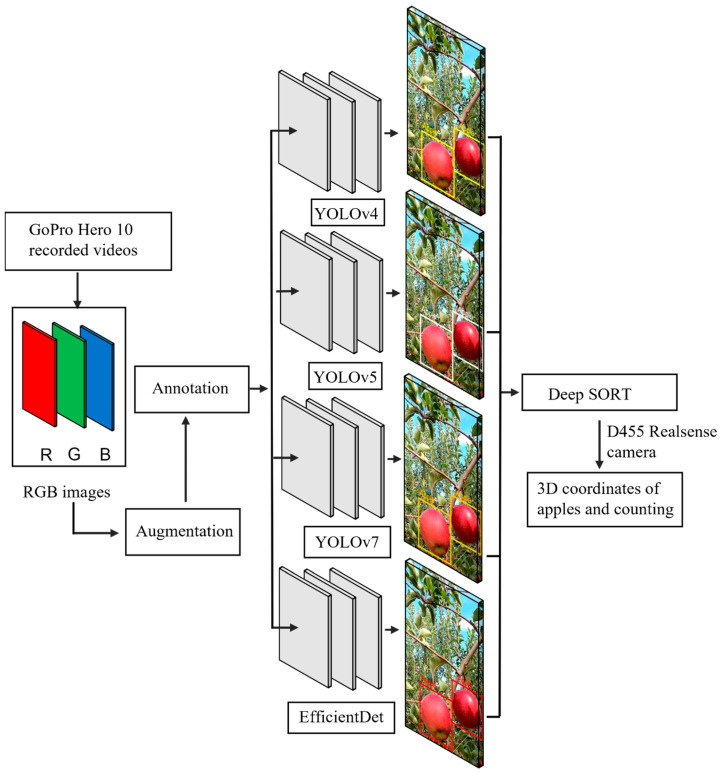
Data augmentation process and training nets for dataset preparation for training and counting using YOLOv4, YOLOv5, YOLOv7, EfficientDet, and Deep SORT.

After annotation, the dataset was split into three groups, training, validation, and testing datasets, with 4500, 1500, and 511 images, respectively. The YOLOv4, YOLOv5, YOLOv7, and EfficientDet one-stage detection neural network models were selected to train the dataset. After training the data, the prediction model files were utilized for real-time detection of apples at different displacement speed levels to compare the accuracy using a Realsense D455 3D camera (**Figure 4**).

### 3.3. Dataset Preparation for Object Detection YOLO

**Figure 5:**
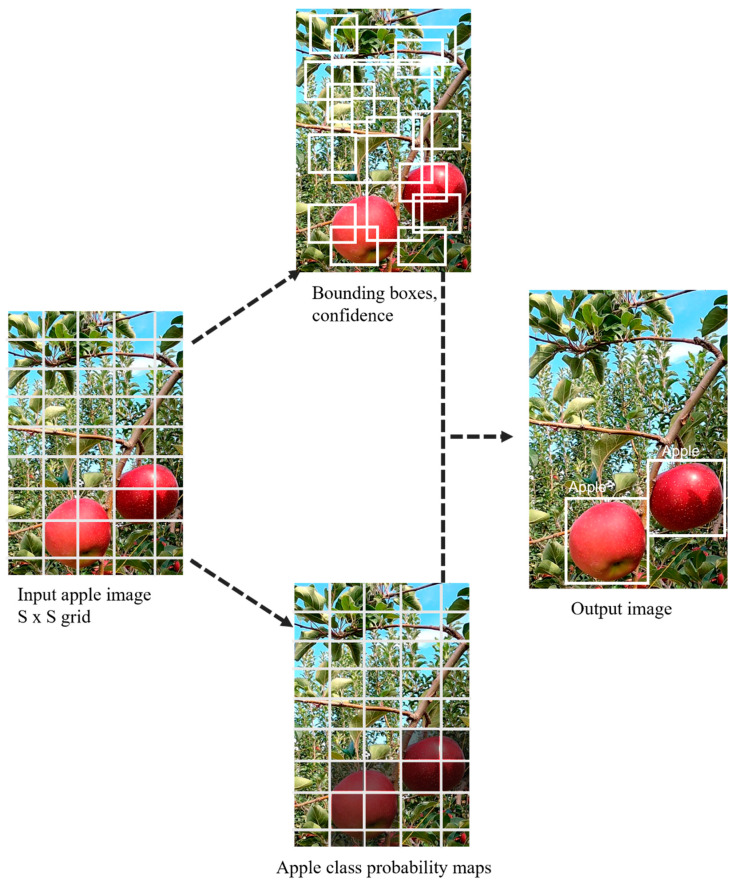
Apple detection based on bounding boxes for the YOLO-based CNN structure.

Object detection algorithms are divided into two categories: two-stage detectors and one-stage detectors. The two-stage detectors identify the target object based on regions of interest and then classify it based on a specific region of interest. YOLO is an anchor-based, one-stage detection algorithm derived from the R-CNN category [29]. YOLO uses a single CNN to localize the image coordinates and to predict the classes via a single pass of the model. Recently, most of the faster and more accurate object detection algorithms have been based on YOLO [30] and have evolved into several versions. The YOLO mechanism works by creating region of interest (ROI) probabilities via regression and adding the S×S grid of the entire image at once. Based on the extracted features (CNN backbones), the ROI determines the target region (**Figure 5**).

YOLOv4 is embedded in the Darknet framework and uses the CSPDarknet-53 CNN to extract features during the training phase and to predict the target object in a given frame. The accuracy of detection in real time depends on the network size, power processing capability, and complexity of the targets. CSPDarknet-53 uses cross-stage partial Darknet-53, which combines an optimal CNN with spatial pyramid pooling (SPP) and a path aggregation network (PANet) to increase the accuracy and speed of detection [31]. Batch normalization combined with high-resolution classifier interrogation with many tuning parameters helps to improve YOLOv4 target detection. Nevertheless, a bag of freebies and a bag of specials increase the algorithm’s performance compared to the previous versions [29].

Ultralytics^®^ YOLOv5 uses the same backbone (CSPDarknet-53) as YOLOv4. However, the PyTorch framework is additionally composed of a feature pyramid network (FPN) combined with a pixel aggregation network (PAN) [32,33]. The main difference is that YOLOv5 replaces the YOLOv3 backbone’s first three layers with a focus layer, which helps reduce not only the impact on mean average precision (mAP) by minimizing the model size by removing some parameter layers but also the floating-point operations per second (FLOPS) at the same time as the CUDA memory [34].

YOLOv7 has outperformed other object-detecting algorithms in terms of having a higher detection accuracy and faster inference speed in real-time object detection tasks and uses a trainable bag of freebies for optimization. Real-time object detection is always challenging even though high-performing computers are available. To effectively utilize the parameters and computation, as a way forward, extension and compound scaling was introduced. The introduced methods for real-time state-of-the-art detection can reduce the number of parameters and computation time by approximately 40% to 50%, respectively. In this study, we compared the accuracy of YOLOv7 in the real-time localization of apples with that of other state-of-the-art detectors [35].

The EfficientDet single-shot detector comprises an EfficientNet backbone to extract features. The neural network model neck contains a bidirectional feature pyramid network (BiFPN), which helps increase the detection efficiency via apple feature fusion with fast normalization. The detected apples are labeled, and the class is predicted by the box prediction net on the model’s head part. Another important feature of this model is scaling, which is a single and compound scaling factor that controls the width, depth, and resolution. All these features help the EfficienDet algorithm perform with high accuracy, and the algorithm was released by Google Research, LLC [36].

### 3.4. Performance Metrics

The goal of the object detection algorithms was to differentiate the apples from the background and localize them since they had a single class: apple. Based on the PASCAL VOC challenge, several metrics were applied to evaluate the performances of the trained model [37]. To compare or to have an indication of the trained model, the intersection over union (IoU), which compares the distance between the prediction box and the ground truth box (labeled bounding box), can be used, and the range can vary from zero to one. Through comparison to IoU, the true-positive (TP), false-positive (FP), and false-negative (FN) detections were defined. Correct detection of apples is the TP; nevertheless, when leaves, barnacles, or any other object was detected as TP apples, this detection is considered a false positive (FP), and false negatives (FN) represent the missing target objects that were not detected by the trained algorithm. In this case, the apples were not detected but should have been. Based on these values of model precision, the effectiveness of the model indicated by the recall and the mean average precision mAP was calculated as the area under the curve, denoted by the precision and recall, where C is the total class number, T is the IoU threshold number, k is the IoU threshold, P(k) is the precision, and R(k) is the recall, using the following expressions.

(1) $$\mathrm{Precision}=\frac{\mathrm{TP}}{\mathrm{TP}+\mathrm{FP}}$$

(2) $$\mathrm{Recall}=\frac{\mathrm{TP}}{\mathrm{TP}+\mathrm{FN}}$$

(3) $$\mathrm{mAP}=\frac{1}{C}\overset{T}{\underset{k=1}{\sum }}P\left(k\right)\Delta R\left(k\right)$$

### 3.5. Dynamic Localization of Apples

After obtaining the trained models, the real-world camera was used to obtain the 2D pixel coordinates on the RGB images based on trained neural networks. Then, after obtaining the 2D coordinates, the geometric center point was calculated. Thus, the 2D pixel coordinates of the apple in the RGB image stream were aligned with the depth image stream to obtain the depth values relative to the real-world camera.

The apple bounding box coordinates in the top left corner are denoted as ($x_{i}$, $y_{i}$), and the width and height of those are denoted as ($x_{j}$, $y_{j}$). The geometric center of the apple (x, y) was calculated using Equations (4) and (5).

(4) $$x=\left(x_{i}+x_{j}\right)/2$$

(5) $$y=\left(y_{i}+y_{j}\right)/2$$

**Figure 6:**
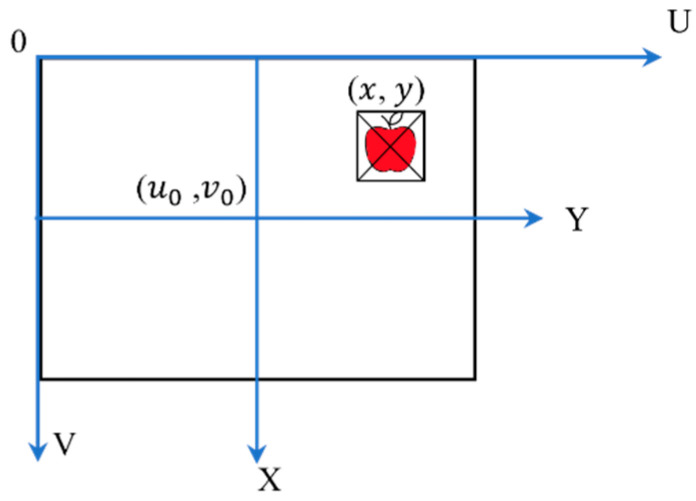
Camera and image coordinates for individual apple localization.

**Figure 7:**
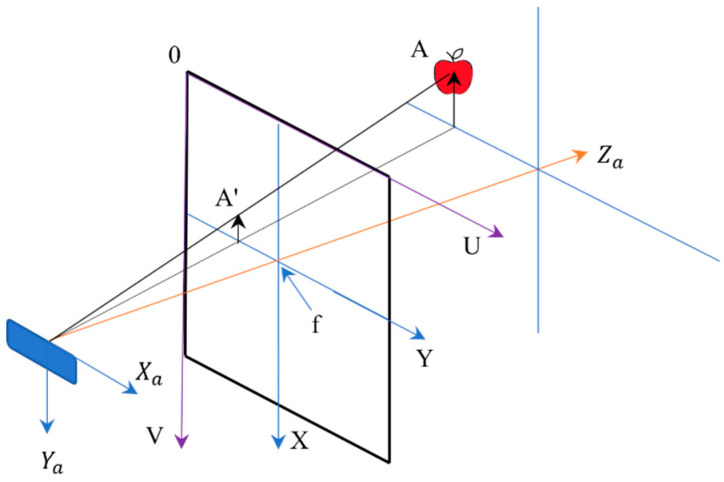
Pixel coordinates vs. image coordinates for individual apple localization.

The apple coordinates in the image coordinate system were obtained using Equation (6), where $d_{x}$ and $d_{y}$ are mm/pixel and the coordinates originate ($u_{0}$, $v_{0}$) from the intersection of the *X* and *Y* main axes in the image coordinate system. The directions are shown in **Figure 6**. The optical center of the depth camera was perpendicular to the *X* and *Y* axes and crossed the image coordinates at the center (**Figure 7**). As indicated, $X_{a}$ and $Y_{a}$ are parallel and $Z_{a}$ is perpendicular to *X* and *Y*, ($X_{a}$, $Y_{a}$, and $Z_{a}$ are in millimeters and perpendicular).

(6) $$\left[\begin{array}{c} u\\ v\\ 1 \end{array}\right]=\left[\begin{array}{ccc} d_{x} & 0 & u_{0}\\ 0 & d_{y} & v_{0}\\ 0 & 0 & 1 \end{array}\right]\left[\begin{array}{c} x\\ y\\ 1 \end{array}\right].$$

The camera coordinate system and image coordinate system have a proportional relationship (Equation (7) according to the camera imaging principle; if the apple coordinates in the camera coordinates are A ($X_{a}$, $Y_{a}$, $Z_{a}$), the pixel position in the RGB image is A’(x, y). Equation (8) can be used to obtain the apple coordinates in the camera coordinate system.

(7) $$\frac{Z_{a}\;}{f}=\frac{X_{a}}{x}=\frac{Y_{a}}{y}$$

(8) $$Z_{a}\left[\begin{array}{c} x\\ y\\ 1 \end{array}\right]=\left[\begin{array}{cccc} f & 0 & 0 & 0\\ 0 & f & 0 & 0\\ 0 & 0 & 1 & 0 \end{array}\right]\left[\begin{array}{c} X_{a}\;\\ Y_{a}\;\\ Z_{a}\;\\ 1 \end{array}\right]$$

Using a software development kit (SDK) real-sense depth camera, the $f/d_{x}$, $f/d_{y}$, $u_{0}$, and $v_{0}$ initial parameters were obtained (f is the camera focal length, and the depth image $Z_{a}$ was obtained for the detected apple). Equations (9) and (10) were applied to obtain $X_{a}$ and $Y_{a}$.

(9) $$X_{a}=\left(x-u_{0}\right)\frac{Z_{a}}{f/d_{x}}$$

(10) $$Y_{a}=\left(y-v_{0}\right)\frac{Z_{a}}{f/d_{y}}$$

### 3.6. Outdoor Experimental Setup as Artificially Built Orchard

**Figure 8:**
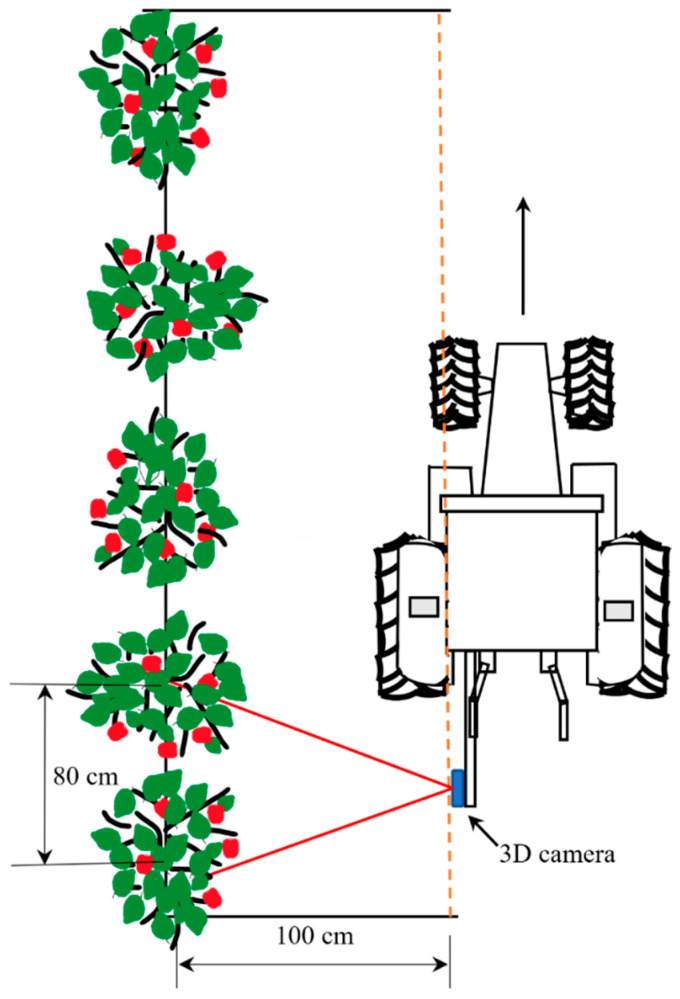
Outdoor data collection procedure at different forward speeds and orientation angles for the recognition of apples and distance information using a 3D camera.

**Figure 9:**
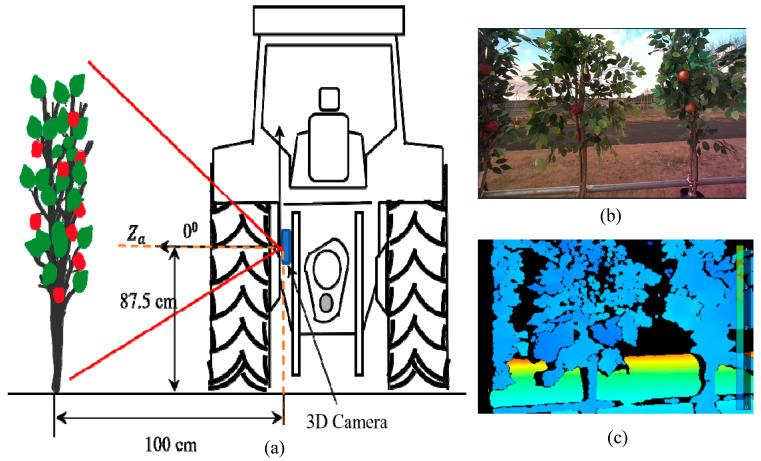
(**a**) A 3D camera at 90° orientation, (**b**) RGB stream corresponding to the 90° orientation, and (**c**) a depth map.

**Figure 10:**
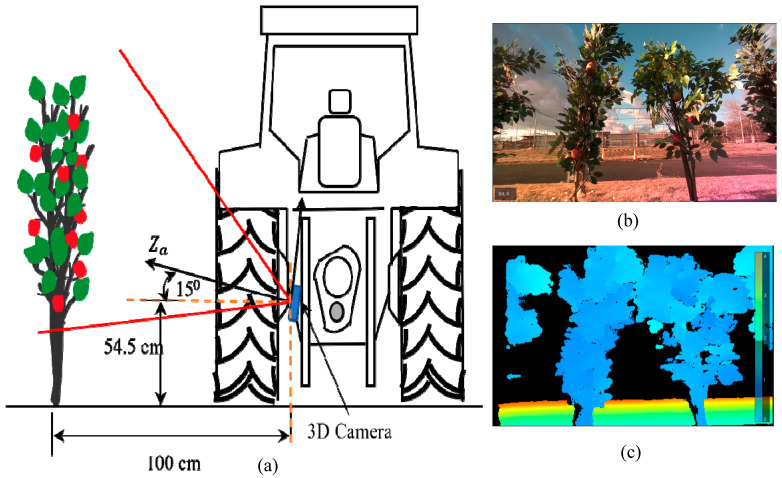
(**a**) A 3D camera at 15° orientation, (**b**) RGB stream corresponding to the 15° orientation, and (**c**) a depth stream.

**Figure 11:**
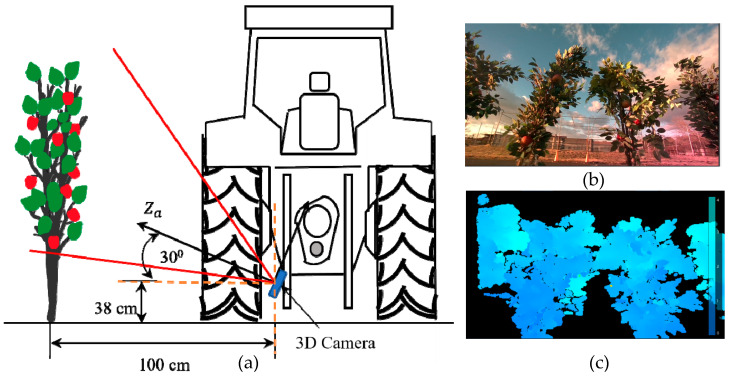
(**a**) A 3D camera at 30° orientation, (**b**) RGB stream corresponding to the 30° camera orientation angle, and (**c**) a depth stream.

**Figure 12:**
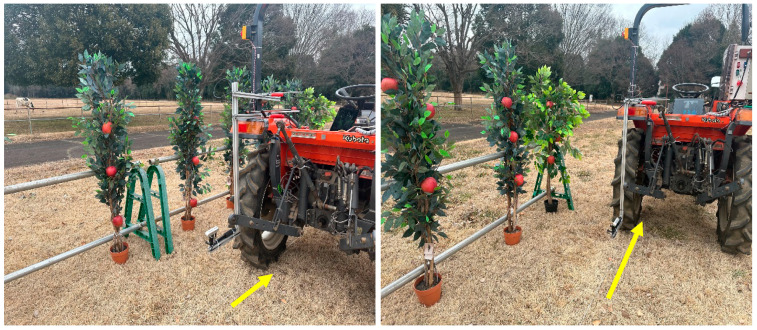
Outdoor experiments using a four-wheel tractor and artificial trees to create a specially designed orchard.

After depth frames were aligned with the RGB frames, testing was carried out to obtain accurate depth values while integrating the trained models. The image frame size from the 3D camera was set to 1280 × 720 pixels. The bounding boxes were plotted inside this frame, wherein the minimum $X_{a}$ and maximum $X_{a}$ ranged from 0 to 1280 pixels, and the minimum $Y_{a}$ and maximum $Y_{a}$ ranged from 0 to 720 pixels. The $Z_{a}$ value was adjusted according to the depth mapping from infrared imaging with the same frame proportion. The experiment was carried out at the Tsukuba-Plant Innovation Research Center, University of Tsukuba, Ibaraki, Japan. A four-wheel tractor (Kubota^®^ KL21) was used to mount the 3D camera at the backend, and 30 real apples were carefully attached to 10 artificial apple trees to create a specially designed orchard to simulate real environmental conditions. Inter-row space was maintained at 80 cm, and the tractor was driven 1 m parallel to the apple trees (**Figure 8**). The 3D camera was placed at three different angles: 0° (perpendicular to the tree row), 15°, and 30° (**Figure 9**, **Figure 10** and **Figure 11**). For each angle, the tractor was driven forward at three speeds: 0.052 ms^−1^, 0.069 ms^−1^, and 0.098 ms^−1^. The selected speeds based on tractor gear 3, 5, and 6 and the rpm of the engine were kept at 1000 during the experiment. A 3D camera was attached to the back of the tractor, which could be adjusted (**Figure 12**).

### 3.7. Experimental Evaluation

**Figure 13:**
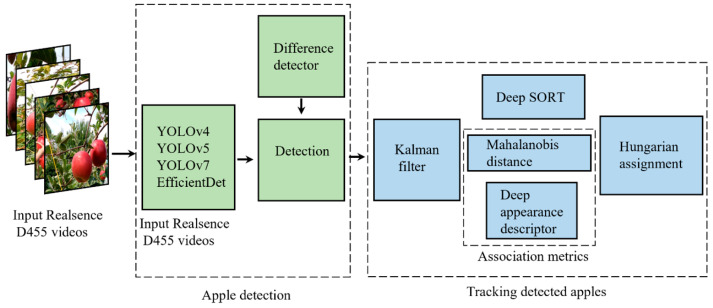
Architecture of the Deep SORT algorithm for tracking detected apples.

Once the apple was detected using deep learning algorithms, the detected apple was tracked until the apple was away from the camera frame. In the operation of harvesting and yield estimation, this step was essential. Without it, the single detected apple in the first frame could have been counted every time the frame changed. Simple online real-time tracking (SORT) is a method of multiple object tracking (MOT) based on spatial and temporal features. Once the object detection algorithm detected the apples in the frame, the estimation of the same apple in the next frame was performed by a constant velocity model via the Kalman filter framework. In data association, the Hungarian algorithm was used to check the IoU of the detection based on a threshold value. In the IoU threshold, the value was set to 50%. In this study, we applied the Deep SORT algorithm for tracking tasks. As an extension of the SORT algorithm, Deep SORT can be applied in CNNs [38]. In addition to the Kalman filter and Hungarian algorithms mentioned before, Deep SORT was applied for the prediction of bunding boxes to extract the localization patterns, meaning that, once the object of interest was localized under a predefined confidence, the localization coordinates were loaded into the CNN embedded in the algorithm source. This technique helps in tracking the apples and maintaining their unique IDs by avoiding occlusion and repetition of IDs among the frames (**Figure 13**). We used MobileNetV2 as a CNN in the Deep SORT algorithm to track the localization of detections. In this way, we could overcome one of the main problems of MOT (multiple object tracking), which is related to a failure to track after the association metrics move too far away from the target.

The real distance values (reference) were manually measured using a measuring tape for 30 apples, and the measurements were taken three times from three angles once the apple tree came to the center position of the camera frame.

**Figure 14:**
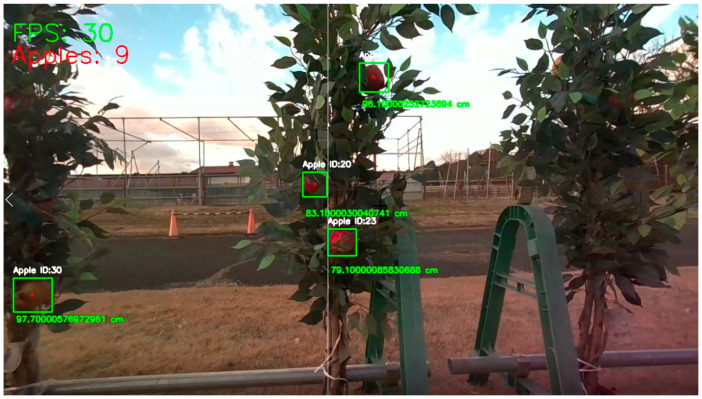
Apple coordinates and counting with the YOLOv5 and Deep SORT algorithms using a vertical ROI line.

The counting was performed after the detected apples passed through a vertical line. The optimal ROI was set to 50%, the middle of the frame, to allow more flexibility for the neural network in localizing the apples, and the camera location represented the middle point (**Figure 14**). The training and deployment of models coupled with the Deep SORT algorithm and a 3D camera were performed using a computer that had a NVIDIA^®^ GTX 1650™, 4 GB GPU, Windows^®^ 10™, 64 bits, 32 GB of RAM with an Intel^®^ Xeon™ E5-1607 processor.

## 4. Results

This study was carried out to compare the dynamic accuracy of YOLOv4, YOLOv5, YOLOv7 and EfficientDet in detecting apples while obtaining the 3D coordinates in real time. The results have important implications for the development of robotics arms operating in real time and determining how much efficiency can be achieved in terms of localization.

### 4.1. Training Model Results

**Figure 15:**
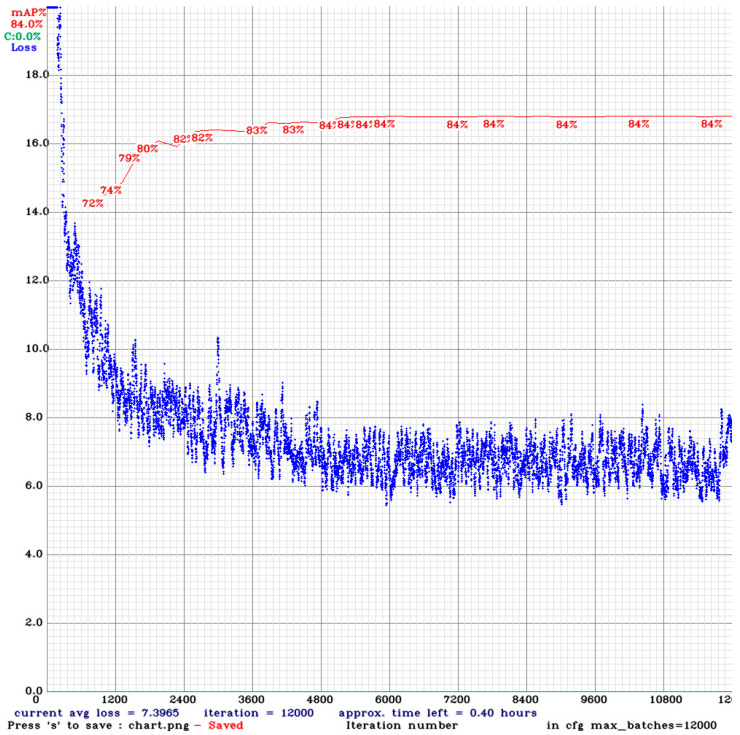
Training performance of datasets using the YOLOv4 algorithm.

The YOLOv4 object detection algorithm was trained under the Darknet framework for apples based on programming languages C and CUDA. The model reached the minimum average loss after 12,000 batch iteration steps (**Figure 15**). The mean average reached 84%, indicating that no overfitting of the model with the training dataset occurred. The training took 72 h. To achieve higher accuracy in the trained models, all the models were trained until they achieved stability of the mAP.

**Figure 16:**
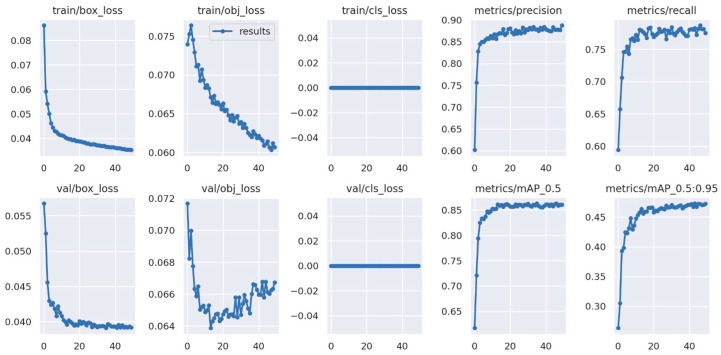
Training performance of datasets using the YOLOv5 algorithm.

YOLOv5s was trained under the PyTorch framework in the Google LLC Colab environment. The epoch size was set to 50, and 4.57 h were required for the completion of the training. The YOLOv5 results were evaluated using the TensorBoard™ interface (**Figure 16**). Through comparison with the YOLOv4 Darknet framework, the TensorBoard™ interface provided a distinct overview of the model. As a supervised learning algorithm, after 40 epochs of training as a learning parameter, the object loss achieved a satisfactory value, and the mean average reached 86%. The precision of the metrics and the mAP values became more stable after 20 epochs, which is consistent with the number of epochs needed to obtain the best results.

**Figure 17:**
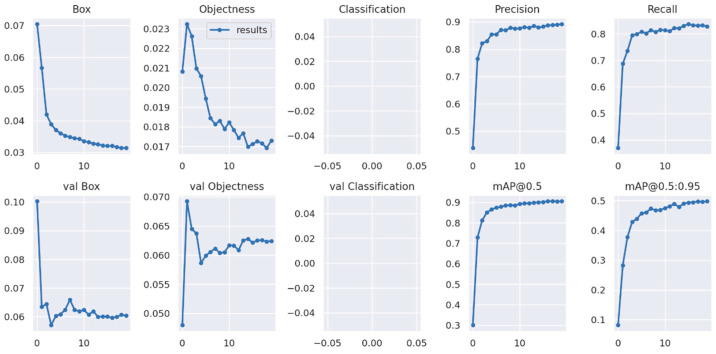
Training performance of datasets using the YOLOv7 algorithm.

**Figure 18:**
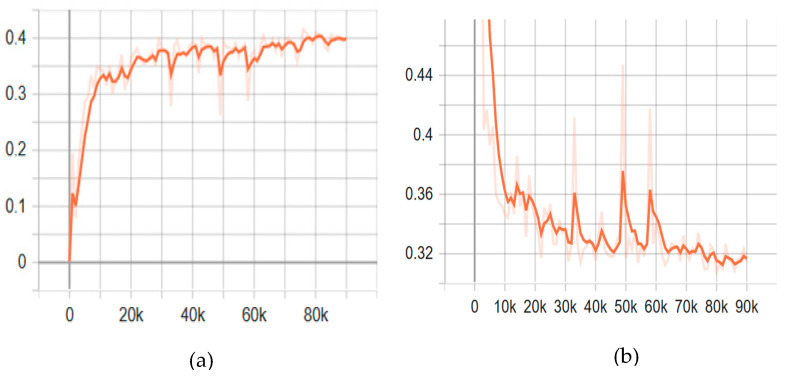
Training performance of datasets using EfficientDet (**a**) precision curve (mAP@0.5) and (**b**) total loss curve.

YOLOv7 was trained in the Google LLC Colab environment under the PyTorch framework, which is similar to YOLOv5. In the YOLOv7 training, 20 epochs took 2.549 h (**Figure 17**). The results show that the mAP@0.5 was 0.905 and became stable after 10 epochs. EfficientDet training was also performed in the computer based on TensorFlow environment. When compared with the YOLO model, the network size of (512 × 512) was a major factor. This model was also a single-shot detector mostly suitable for detecting small objects. The training results were obtained after 60,000 steps, and the mAP@0.5 was 0.775 (**Figure 18**). For the training of each DNN model, the same augmented, annotated, and labeled dataset was used (**Table 1**).

### 4.2. Real-Time Dynamic Detection Results

**Figure 19:**
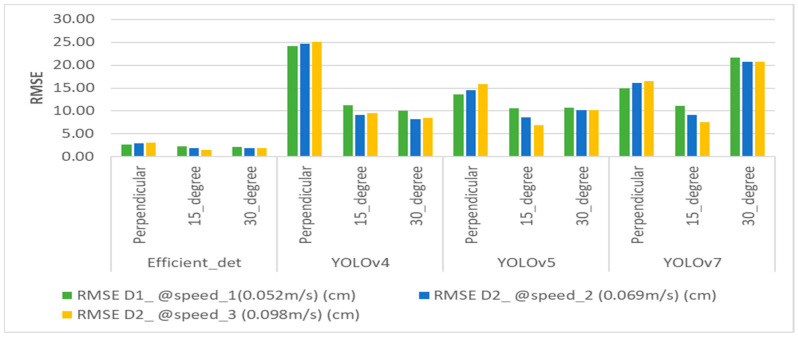
Summary of RMSE values for different orientation angles and deep learning algorithms.

**Figure 20:**
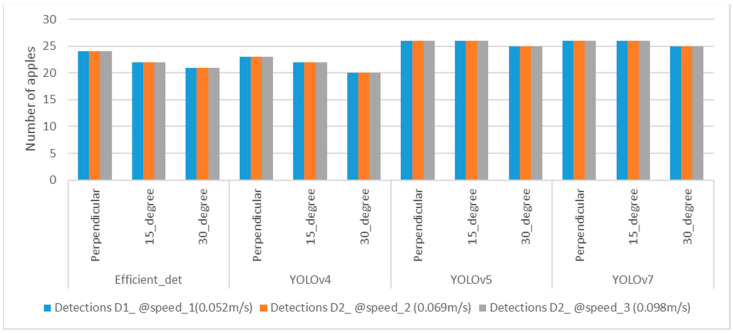
Number of apples detected out of 30 for different orientation angles and deep learning algorithms.

The real-time dynamic accuracy and counting of apples were determined based on DNNs followed by Deep SORT tracking. The distances from the 3D camera to the apples were manually measured from three angles for 30 apples and compared to the manual distance with dynamic detection for three speed values. The root mean square error (RMSE) values for each observation were calculated, and the error bars are also reported in the graphs (Appendix A). Based on the RMSE values, EfficientDet at an orientation angle of 15° with a speed of 0.098 ms^−1^ provided the most accurate distances than the other models and angles (**Figure 19**). In terms of counting, the highest detection accuracy was obtained by YOLOv5 and YOLOv7 for 30 apples distributed randomly across the different artificial trees (**Figure 20**).

## 5. Discussion

This study focused on developing an apple recognition and counting system using deep learning algorithms based on the Realsense D455 camera and integrated with Deep SORT, in specially designed orchard datasets for robotic arm applications. Conventional apple orchard systems are complex, making it difficult to operate machineries enabling precision agriculture practices. In Japan, the Apple Research Centre in Aomori Prefecture developed spindle-type apple orchard systems that can be used for robotic management practices. In this study, we developed similar spindle-type orchard conditions at the Tsukuba Plant Innovation Research Center (TPIRC, University of Tsukuba). In the artificial trees of the specially designed orchard, we randomly attached real apples to conduct the experiments using a 3D camera and a tractor to study robotic arm deployment at the 3-point hitch position with an autonomous tractor.

Three-dimensional cameras have the ability to provide depth values and RGB information using DNNs based on RGB frame objects that can be localized in X and Y coordinates referenced to the pixel values in the frame. Once the depth frames aligned with the corresponding RGB values, the distance values were obtained for the detected apples. The developed DNNs can be used for detecting objects based on the training dataset. For these experiments, YOLOv4, YOLOv5, YOLOv7, and EfficientDet were employed. In this study, we used one-stage detection networks, which can achieve higher detection accuracy in fruit detection [39]. The aim of using these DNNs in outside conditions was to analyze their dynamic accuracy in apple detection as well as to determine the optimal distance between the camera and the detected apples for robotic arm manipulations in real time for spindle-type orchards.

Even in recently developed spindle-type orchard systems, noise caused by occlusions by leaves and light variation still hinders performance, while wind makes this environment even more complex. The variation in light, shadows, and movement of trees and leaves cause errors in detection as well as depth values. The apple counting results show the effect of occlusion due to leaves and wind-induced leaf movement on missed detection of apples. Thus, the abovementioned detection errors may create problems in robotic arm operations as well as in the counting of apples.

Dynamic accuracy in the detection of apples’ distance to the center position was compared at three different speeds and three different angles in specially designed spindle-type apple orchard conditions. EfficientDet, at the 15° orientation camera position, provided more accurate *Z*-axis distance values, which means that in real time, the speed of the robotic arms can be operated with additional support to eliminate errors. In terms of the number of detections achieved, YOLOv5 and YOLOv7 showed a higher number of detections, achieving a maximum counting accuracy of 86.6%. Based on the results, an EfficientDet- based vision system manipulator would have enough flexibility in the gripping system to achieve maximum harvesting efficiency with an error of 1.54 cm.

Additional support can be given to reduce noise, and the variation in light can be reduced by providing extra light before starting the vision system before each detection. Furthermore, the detection can be performed in two stages using two cameras: one camera can be mounted on the base of the manipulator, and the other camera can be mounted near the gripper. Moreover, a sensor making additional depth measurements could be aligned with the 3D camera to increase the accuracy of depth measurements. The other option is to detect the same apple after the manipulator is halfway through its forward-moving cycle, which may result in more precise gripping compared to one-time detection.

## 6. Conclusions

This study was carried out to compare the dynamic accuracy of YOLOv4, YOLOv5, YOLOv7, and EfficientDet and state-of-the-art, one-stage detection algorithms in detecting and counting apples and to determine the accuracy of the forward-moving vision system in apple recognition and localization while deploying a robotic arm or an end effector to harvest apples. Apple harvesting in a specially designed orchard via a robotic system was considered to prevent mechanical damage and aid accurate detection and obtention of the 3D coordinates of apples for grasping apples from trees. The Realsense 3D camera was selected to develop the forward-moving mechanism on-board a four-wheel tractor by keeping the camera angles at perpendicular, 15°, and 30° orientations to the apple trees. This improvement ensures that apples can freely fall into the collector via a conveyer after the gripper harvests apples from the trees. The results show that YOLOv4 had less accuracy based on the RMSE when the camera was perpendicular at three dynamic speeds. EfficientDet had fewer errors for the three camera orientations at the three speeds and had the RMSE of less than 3 cm. Based on the RMSE values, it can be concluded that EfficientDet at 15° had more accurate *Z*-axis distance recognition accuracy, while in terms of counting, YOLOv5 and YOLOv7 outperformed other models in real time under architecturally facilitated, outdoor conditions. The developed vision system can be employed for robotic arm manipulations with additional light support while performing operations such as accurate grasping of apples in a specially designed orchard.

## Figures and Tables

**Table 1:** Performance evaluation summary of training datasets for apple detection models.

Model.	Precision	Recall	F1	mAP @0.5
**YOLOv4**	0.840	0.790	0.810	0.840
**YOLOv5**	0.874	0.783	0.830	0.861
**YOLOv7**	0.892	0.828	0.860	0.905
**EfficientDet**	0.950	0.950	-	0.775

**Figure A1:**
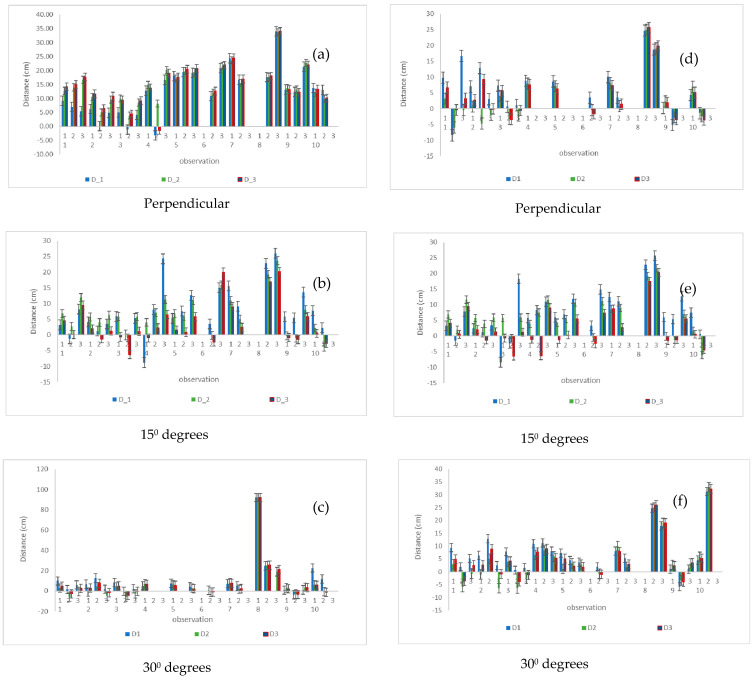

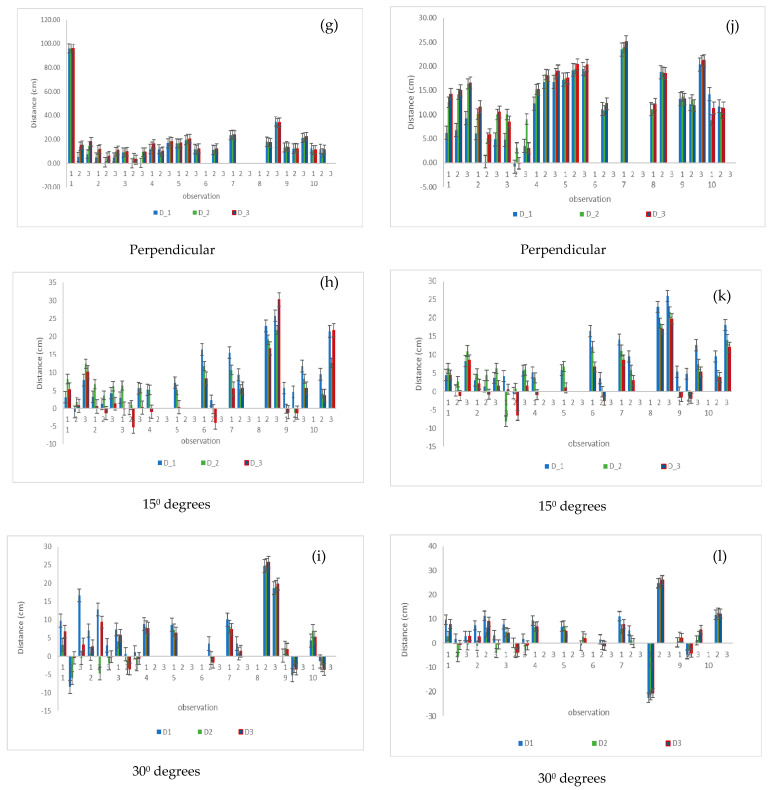
Difference between the real distance and the measured distance at three different speeds and three different angles. (**a**–**c**): YOLOv7 Deep SORT; (**d**–**f**): YOLOv5 Deep SORT; Difference between the real distance and the measured distance in three different speeds and three different angles. (**g**–**i**): YOLOv4 Deep SORT; (**j**–**l**): EfficientDet Deep SORT; D1, D2, D3 are difference between the measured depths value and the 3D-camera-detection depths value at speed of 0.052 ms^−1^, 0.069 ms^−1^ and 0.098 ms^−1^ respectively.

## Data Availability

The dataset that was generated and analyzed during this study is available from the corresponding author upon reasonable request, but restrictions apply to the data reproducibility and commercially confident details.
